# Clinical relevance of the lnc‐HNF1B‐3:1 genetic polymorphisms in Western Chinese tuberculosis patients

**DOI:** 10.1002/jcla.23076

**Published:** 2019-11-06

**Authors:** Qian Wu, Huiyu Zhong, Hao Bai, Tangyuheng Liu, Jiajia Song, Yang Wen, Xingbo Song, Binwu Ying

**Affiliations:** ^1^ Department of Laboratory Medicine West China Hospital Sichuan University Chengdu P. R China

**Keywords:** adverse drug reactions, anti‐tuberculosis treatment, lnc‐HNF1B‐3:1, single nucleotide polymorphism, tuberculosis

## Abstract

**Background:**

Tuberculosis remains a global public health problem. Genetic polymorphisms may affect the susceptibility, clinical characteristics, and adverse drug reactions of patients with TB. The present study aimed to examine the association of single nucleotide polymorphisms of lncRNA‐HNF1B‐3:1 with the clinical manifestation of TB in a Western Chinese population.

**Method:**

A total of 526 tuberculosis patients and 561 healthy subjects were recruited in Western China. The correlation between lnc‐HNF1B‐3:1 polymorphism and tuberculosis susceptibility was investigated. Moreover, the influence on adverse drug reactions following treatment was explored. A total of 7 SNPs within the lnc‐HNF1B‐3:1 locus was genotyped by the improved multiplex ligation detection reaction method.

**Results:**

No significant associations were noted between TB susceptibility and the presence of all 7 SNPs of the lnc‐HNF1B‐3:1 as determined by single‐locus analysis (All *P* > .05). The AA genotype of rs12939622 (in the dominant model) and the AA genotype of rs4262994 (in the recessive model) caused increased susceptibility of the subjects to fever (*P* < .001 and *P* = .008, respectively). The Rs2542670 G allele was associated with increased risk of thrombocytopenia, leukopenia, and chronic kidney damage following drug administration (*P* = .007, .029, .003, respectively).

**Conclusion:**

The present study reported for the first time that the rs12939622, rs4262994 and rs2542670 genotypes in lnc‐HNF1B‐3:1 locus may influence the clinical manifestations of tuberculosis.

## 1 INTRODUCTION

Tuberculosis (TB) is an ancient human disease that may have evolved with modern human populations over thousands of years.^1^ It is still one of the top ten leading causes of death worldwide. The World Health Organization reported that approximately 10 million new TB cases have emerged that were equivalent to 133 cases per 100 000 population in 2017. China is one of the top 20 TB countries with the second largest number of new cases in 2017 over the world. It is interesting to note that approximately 23% of the world population has been exposed to *Mycobacterium tuberculosis* (MTB). These subjects have a high risk of developing active TB during their lifetime.^2^ Only a small proportion of the subjects with latent tuberculosis infection (LTBI) develop clinical tuberculosis. Epidemiological and genetic studies have demonstrated that human genetic factors have a significant impact on this interindividual difference, although the exact molecular mechanisms of this disease remain unclear.^3^

Sequencing efforts have revealed several thousands of long non‐coding RNAs (lncRNAs) produced from the human genome, which are longer than 200 nucleotides and have no apparent protein‐coding potential.^4^ LncRNAs are found in different cellular compartments and have the ability to determine the macromolecular architectures, such as nuclear paraspeckles.^5^ Previous studies have suggested that lncRNAs can participate in gene expression and play an important role in numerous biological processes, such as cell differentiation, cell cycle, and epigenetic regulation.^6^, ^7^ Several hundreds of thousands of annotated human lncRNAs have been identified in the latest version of the LNCipedia.^8^ Only a limited number of these annotated lncRNAs have functional characterization, while the vast majority of them possess unknown functions. Lnc‐HNF1B‐3:1(ENSG00000250985) is located in chr17:37293561‐37852819 and has a length of 2,229 nucleotides. Its exact function has not been investigated to date.

With the continuous improvement of high‐throughput genotyping and sequencing technologies, genome‐wide association studies (GWAS) have enabled in the past decades the identification of the genetic factors responsible for the development of complex diseases.^9^ In addition, approximately half of the identified disease‐associated single nucleotide polymorphisms (SNPs) do not include protein‐coding genes.^10^ In 2012, Thye et al^11^ reported a novel association between the rs2057178 polymorphism of chromosome 11p13 with resistance to TB. Another study demonstrated a link between TB and the variants located in the *ASAP1* gene in European subjects.^12^ Notably, the SNPs located in the lncRNAs could also influence the occurrence and development of the diseases. A recent study identified that the rs920778 polymorphism could regulate the expression levels of the lncRNA HOTAIR in esophageal squamous cell carcinoma via a novel intronic enhancer.^13^ A multi‐center study confirmed that rs6983267 and its accompanying lncRNA CCAT2 were able to induce myeloid malignancies due to unique SNP‐specific RNA mutations.^14^ Accumulating evidence indicates that lncRNA polymorphisms may be potential novel biomarkers used for diagnosis, therapy, and prognosis of human diseases.

The two major challenges faced by several medical practitioners are the diagnosis of TB and the incidence of adverse drug reactions (ADRs) caused by treatment against TB. Notably, the side effects are the leading cause (57%) of unsuccessful response to TB treatment of patients in the Centers for Disease Control in China.^15^ Therefore, it is of great importance to investigate the susceptibility of the SNP loci that may be involved in the development of ADRs. In the present study, we genotyped 7 SNPs within lnc‐HNF1B‐3:1 among 526 tuberculosis cases and 561 healthy subjects in order to analyze the association between lnc‐HNF1B‐3:1 polymorphisms and the clinical characteristics of active tuberculosis patients.

## 2 MATERIALS AND METHODS

### 2.1 Study design and participants

The present study recruited 526 TB cases and 561 healthy controls. The cases were enrolled from West China hospital outpatients or inpatients between November 2014 and September 2016. TB diagnosis was based on typical symptoms, radiological evidence of active TB and microbiological findings. The diagnosis was confirmed by two experienced respiratory physicians. All TB patients were treated by a course of 6‐month chemotherapy including isoniazid (INH), rifampicin (RIF), pyrazinamide (PZA), and ethambutol (EMB). Patients with hepatitis, HIV infection, other infectious diseases, cancers, pregnancy and cardiac, hematological, and renal diseases were excluded. In addition, the patients with specific liver and kidney abnormalities and hematological abnormalities prior to anti‐tuberculosis treatment were also excluded. Healthy controls were enrolled from the Physical Examination Center of the West China Hospital. These subjects were examined clinically and revealed to be negative for sputum smear testing. The control subjects were matched with TB patients in age and gender. All participants were non‐relatives and informed consent was provided for their participation in the study. The study was approved by the Ethics committee of the West China Hospital of Sichuan University.

### 2.2 Clinical phenotypes

Demographic and clinical data of the study population were accessed from the Hospital Information System of the West China Hospital of the Sichuan University. The blood samples were obtained from each participant for genotyping. Pre‐therapeutic laboratory tests and chest X‐ray examinations were achieved in the clinical laboratory of the West China Hospital. The patients were followed up for at least 6 months during the course of chemotherapy to assess ADRs and were examined by laboratory tests monthly. In the present study, the anti‐tuberculosis drug (ATD)‐induced ADRs included hematological toxicity, hepatotoxicity, and renal damage. The main criteria for ATD‐ADRs were as follows: (a) anemia, which was defined as hemoglobin ≤80 g/l; (b) Leukopenia, which was defined as leukocyte counts <2.0 × 10^9^/L; (c) Thrombocytopenia, which was defined as platelet counts <75 × 10^9^/L; (d) Hyperbilirubinemia, which was referred to an increase of total bilirubin ≥1.5 times of the upper limit of the normal levels (42 μmol/L)^16^; (e) AST and ALT levels higher than and/or equal to (≥) 3 times of the upper limit of the normal levels (120 IU/L) with hepatitis symptoms, or ALT and AST levels ≥5 times of the upper limit of normal levels (150 IU/L) without symptoms, which were considered as ATD‐induced hepatotoxicity (ATDH)^17^, ^18^; (e) Acute kidney injury (AKI) defined as a sudden decline in renal function, such as a 1.5‐fold increase in serum creatinine (Scr) from the reference within 7 days; (f) Chronic kidney disease (CKD) present in kidney damage or glomerular filtration rate (GFR) <60 mL/min/1.73 m^2^ for more than 3 months.^19^

### 2.3 Single nucleotide polymorphism selection and genotyping

The genetic polymorphism data of lnc‐HNF1B‐3:1 were obtained from 1000 Genomes (https://www.1000genomes.org/index.html) and the dbSNP database (https://www.ncbi.nlm.nih.gov/snp). SNPs were included if they were located in potential functional regions (intron, promoter, and untranslated region) and were an optimal representation of the Beijing Han population with a small allele frequency (MAF) >0.05. Ultimately, seven SNPs (rs2542670, rs1051838, rs1416, rs4262994, rs12939622, rs2688, and rs8075185) were selected and successfully genotyped in the present work. The QIAamp DNA blood mini kit (Qiagen) was used to extract genomic DNA from the peripheral blood using the following methodology: The DNA samples were stored at −80°C. An improved multiplex ligation detection reaction (iMLDR) method (Genesky Biotechnologies Inc) was used for genotyping. ddH_2_O was used as a negative control for each reaction. Approximately 10% of the samples were selected for repeated genotyping and the results revealed no difference.

### 2.4 Statistical analysis

The Hardy‐Weinberg Equilibrium (HWE) was employed for cases, whereas the Goodness‐of‐fit Chi‐square test was used for control subjects (χ^2^). The differences of the demographic data were assessed by the chi‐square test (for categorical variables) or Student's *t* test (for continuous variables). The differences in the genotype frequencies and allele frequencies of the SNPs between cases and controls were estimated by the Pearson chi‐square test. The strength of association was estimated by the odds ratio (OR). The 95% confidence intervals (CIs) were estimated by logistic regression analyses. All ORs were adjusted by age and gender. All the statistical analyses were two‐sided and a *P* < .05) was set as a criterion for significant differences. All statistical analyses were employed by the SPSS statistical software (version 22.0; SPSS Inc).

## 3 RESULTS

### 3.1 General characteristics of the study subjects

The demographic and clinical characteristics of the study participants are presented in Table 1. The average age and sex exhibited no significant differences between TB and healthy subjects (HC) (*P* = .254 and .385, respectively). The TB groups exhibited a lower body mass index (BMI) (20.46 vs 23.53 kg/m^2^, *P* < .001), a higher rate of BCG scar (52.85% vs 42.78%, *P* < .001) and a higher proportion of smoking subjects (57.22% vs 39.93%, *P* < .001) compared with the corresponding parameters noted in the HC. TB cohorts were divided into three subgroups, including pulmonary tuberculosis (PTB, 51.14%), extra‐pulmonary tuberculosis (EPTB, 11.41%), and pulmonary tuberculosis combined with extra‐pulmonary tuberculosis (PTB & EPTB, 37.45%). In addition, basic laboratory examinations of all participants and computed tomography (CT) of cases were performed prior to treatment. The TB group exhibited lower levels of albumin and hemoglobin and higher levels of platelets, erythrocyte sedimentation rate (ESR), leukocytes, and monocytes compared with those of the HC group (all *P* < .001). Among the TB patients, the positive rate of TB‐DNA was the highest (36.48%). Lower percentages were noted for the smear microscopy (30.63%) and culture (10.08%). With regard to the results of CT, it was concluded that the common manifestations of TB were infiltration and effusion, fibrosis and calcification and caseation and cavitation.

**Table 1:** Demographic and clinical data of study participants

Characteristics	TB (n = 526)	HC (n = 561)	*P*
General data			
Age, mean ± SD (y)	42.56 ± 19.23	44.23 ± 11.49	.254
Male/female	324/202	331/230	.385
BMI (kg/m^2^)	20.46 ± 2.58	23.53 ± 2.31	**<.001**
BCG scar n (%)			
Yes	278 (52.85)	240 (42.78)	**<.001**
No	199 (37.83)	250 (44.56)	
Unknown	49 (9.32)	71 (12.66)	
Smoking n (%)			
Smoking	301 (57.22)	224 (39.93)	**<.001**
Ever smoking	100 (19.01)	95 (16.93)	
Nonsmoking	125 (23.76)	242 (43.14)	
TB subtype n (%)			
PTB	269 (51.14)	**—**	**—**
EPTB	60 (11.41)	**—**	**—**
PTB & EPTB	197 (37.45)	**—**	**—**
Laboratory examinations			
Albumin (g/L)	35.53 ± 6.62	46.72 ± 2.60	**<.001**
Erythrocyte (×10^12^/L)	4.9 ± 1.4	4.85 ± 0.46	**<.001**
Hemoglobin (g/L)	121.99 ± 2 5.07	147.26 ± 15.13	**<.001**
Platelets (×10^9^/L)	250.77 ± 67.18	171.12 ± 49.07	**<.001**
Leukocytes (×10^9^/L)	8.57 ± 3.01	6.11 ± 1.30	**<.001**
Monocytes (×10^9^/L)	0.75 ± 0.76	0.35 ± 0.12	**<.001**
ESR (mm/h)	44.00 (18.00‐73.00)	5.43 (1.79‐18.42)	**<.001**
C‐reactive protein (mg/L)	16.8 (4.17‐61.6)	**—**	**—**
Positive TB‐DNA n (%)	178 (36.48)	**—**	**—**
Positive smear n (%)	155 (30.63)	**—**	**—**
Positive culture n (%)	42 (10.08)	**—**	**—**
Main alteration of CT n (%)			
Infiltration and effusion	227 (43.16)	**—**	**—**
Caseation and cavitation	108 (20.53)	**—**	**—**
Fibrosis and calcification	108 (20.53)	**—**	**—**
Proliferation and consolidation	75 (14.26)	**—**	**—**
Normal	8 (1.53)	**—**	**—**

Note

*P* significant associations were denoted in bold.

Abbreviations: TB, tuberculosis; HC, healthy controls; PTB, pulmonary tuberculosis; EPTB, extra‐pulmonary tuberculosis; PTB & EPTB, pulmonary tuberculosis combined with extra‐pulmonary tuberculosis.

### 3.2 Genotype distributions of the lnc‐HNF1B‐3:1 polymorphisms

The seven SNPs were successfully genotyped in 526 cases and 561 controls. The genotype distributions of the 7 SNPs within the *RP11‐37B‐3:1* gene in the control group were in line with Hardy‐Weinberg equilibrium (HWE) (*P* > .05 for all loci). As depicted in Table 2, the alleles and genotypic distribution between TB cases and healthy controls were not significantly different. The association between disease susceptibility and the presence of candidate SNPs was analyzed by an inheritance model that contained the additive, dominant, and recessive models. The results are described in Table 3. Rs2688 seemed to have association with a reduced risk for TB in the additive model (TT vs TG vs GG), with an estimated OR of 0.84 (95% CI = 0.71‐0.99, *P* = .049 following adjustment for age and gender). The data did not exhibit significant differences following Bonferroni correction (*P* = .343). The other 6 SNPs did not show significant differences in the genetic model.

**Table 2:** Genotype distributions of *lnc‐HNF1B‐3:1* polymorphisms of TB patients

SNP		Case n (%)	Control n (%)	OR (95% CI)	*P*	P*		Case n (%)	Control n (%)	*P*
rs2542670	G	299 (28.42)	320 (28.52)	0.99 (0.83‐1.20)	.960	—	GG	46 (8.75)	46 (8.27)	.888
A > G	A	753 (71.58)	802 (71.48)				GA	207 (39.35)	228 (41.01)	
							AA	273 (51.90)	287 (51.62)	
rs1051838	G	464 (44.11)	499 (44.47)	0.98 (0.83‐1.17)	.863	—	GG	108 (20.53)	104 (18.71)	.297
A > G	A	588 (55.89)	623 (55.53)				GA	248 (47.15)	291 (52.34)	
							AA	170 (32.32)	166 (29.86)	
rs1416	T	431 (40.97)	454 (40.46)	1.02 (0.82‐1.21)	.810	—	TT	89 (16.92)	91 (16.37)	.953
C > T	C	621 (59.03)	668 (59.54)				CT	253 (48.10)	272 (48.92)	
							CC	184 (34.98)	198 (35.61)	
rs4262994	A	496 (47.15)	512 (45.63)	1.06 (0.90‐1.26)	.479	—	AA	122 (23.19)	121 (21.76)	.777
C > A	C	556 (52.85)	610 (54.37)				CA	252 (47.91)	270 (48.56)	
							CC	152 (28.90)	170 (30.58)	
rs12939622	G	357 (33.94)	374 (33.33)	1.03 (0.86‐1.23)	.767	—	GG	58 (11.03)	71 (12.77)	.311
A > G	A	695 (66.06)	748 (66.67)				GA	241 (45.82)	232 (41.73)	
							AA	227 (43.16)	258 (46.40)	
rs2688	G	411 (39.07)	485 (43.23)	0.84 (0.71‐0.99)	.049	.343*	GG	80 (15.21)	105 (18.88)	.144
T > G	T	641 (60.93)	637 (56.77)				GT	251 (47.72)	275 (49.46)	
							TT	195 (37.07)	181 (32.55)	
rs8075185	T	519 (49.33)	530 (47.24)	1.09 (0.92‐1.29)	.328		TT	137 (26.05)	128 (23.02)	.462
C > T	C	533 (50.67)	592 (52.76)				CT	245 (46.58)	274 (49.28)	
							CC	144 (27.38)	159 (28.60)	

Note

*P*: *P* value was calculated by Chi‐square test.

P*: *P* value after Bonferroni correction.

**Table 3:** Comparison of lnc‐HNF1B‐3:1 polymorphisms in relation to TB risk

SNP	Additive model			Dominant model		Recessive model	
	OR (95% CI)	*P*	P*	OR (95% CI)	*P*	OR (95% CI)	*P*
rs2542670 A > G	0.99 (0.83‐1.19)	.960	—	0.97 (0.77‐1.23)	.807	1.07 (0.70‐1.65)	.747
rs1051838 A > G	0.99 (0.83‐1.17)	.863	—	0.88 (0.68‐1.14)	.331	1.14 (0.84‐1.53)	.407
rs1416 C > T	1.02 (0.86‐1.21)	.810	—	1.01 (0.79‐1.30)	.914	1.05 (0.76‐1.45)	.757
rs4262994 C > A	1.06 (0.90‐1.25)	.486	—	1.07 (0.82‐1.39)	.612	1.10 (0.59‐1.47)	.341
rs12939622 A > G	1.03 (0.86‐1.22)	.455	—	1.12 (0.88‐1.43)	.347	0.86 (0.59‐1.24)	.407
rs8075185 C > T	1.08 (0.92‐1.28)	.339	—	1.05 (0.84‐1.37)	.723	1.19 (0.90‐1.57)	.216
rs2688 T > G	0.84 (0.71‐0.99)	**.049**	.343*	0.81 (0.63‐1.04)	.096	0.78 (0.57‐1.07)	.125

Note

*P*: *P* value was calculated by Chi‐square test.

P*: *P* value after Bonferroni correction.

### 3.3 Association of lnc‐HNF1B‐3:1 polymorphisms and clinical traits of TB

To further explore the association between genetic variants and clinical features, we collected data from clinical characteristics (fever, weight loss, night sweat, poor appetite, and fatigue), laboratory tests (hepatic, renal, and hematological examinations), and CT scans of the cases. Fever is one of the most common signs of tuberculosis. The data indicated that patients with the AA genotype of rs12939622 (in the dominant model) and the AA genotype of rs4262994 (in the recessive model) appeared more susceptible to fever development (*P* < .001 and *P* = .008, respectively, as shown in Tables 4 and 5). In addition, the wild AA genotype exhibited higher levels of albumin and hemoglobin compared with the mutant genotype (GA+GG genotype) of rs2542670 (*P* = .034 and .036, respectively) in the dominant model (Table 6). However, no statistical evidence of associations between the remaining SNP loci and the clinical features of tuberculosis were observed (data not shown).

**Table 4:** Association of rs12939622 with manifestations of TB patients in the dominant model

Manifestations n (%)	GG + GA (N = 299)	AA (N = 227)	*P*	P*
Fever	140 (48.6)	140 (62.8)	**.001**	**.000**
Weight loss	122 (42.4)	89 (39.9)	.720	
Night sweat	94 (32.6)	77 (34.5)	.573	
Poor appetite	124 (43.1)	98 (43.9)	.722	
Fatigue	109 (37.8)	70 (31.4)	.194	

Note

P* value has been adjusted for logistic regression.

**Table 5:** Association of rs4262994 with manifestations of TB patients in the recessive model

Manifestations n (%)	CC + CA (N = 374)	AA (N = 152)	*P*	P*
Fever	185 (51.1)	95 (63.8)	**.007**	**.008**
Weight loss	145 (40.1)	66 (44.3)	.328	
Night sweat	114 (31.5)	57 (38.3)	.125	
Poor appetite	153 (42.3)	69 (46.3)	.381	
Fatigue	133 (36.7)	46 (30.9)	.265	

Note

P* value has been adjusted for logistic regression.

**Table 6:** Association of rs2542670 with examinations of TB patients in the dominant model

Examinations	GG + GA (N = 253)	AA(N = 273)	*P*
Alb (g/L)	35.5 (30.2‐39.5)	36.95 (31.55‐41.00)	**.034**
Leukocytes (×10^9^/L)	6.52 (4.98‐8.88)	6.48 (4.99‐8.71)	.713
Monocytes (×10^9^/L)	0.41 (0.27‐0.64)	0.42 (0.28‐0.60)	.849
Erythrocytes (×10^12^/L)	4.21 (3.67‐4.77)	4.40 (3.82‐4.79)	.088
Hemoglobin (g/L)	119.5 (101.75‐133)	123.5 (105‐138.75)	**.036**
PLT (×10^9^/L)	229.50 (156.00‐326.5)	230.50 (69.00‐331.50)	.614
CRP (mg/L)	19.10 (3.61‐72.52)	15.10 (4.44‐49.35)	.138
ESR (mm/h)	45.00 (17.00‐73.00)	42.5 (18.00‐72.75)	.551
Positive TB‐DNA n (%)	89 (35.18)	89 (32.60)	.348
Positive smear n (%)	77 (30.43)	78 (28.57)	.631
Positive culture n (%)	17 (6.72)	22 (8.06)	.619

### 3.4 Association of lnc‐HNF1B‐3:1 polymorphisms and ATD‐ADRs

SNPs can affect individual differences in adverse reactions following drug administration. In order to further explore the potential association of the lnc‐HNF1B‐3:1 polymorphisms and the incidence of ATD‐ADRs, we investigated 7 common drug adverse reactions following anti‐TB treatment, including hematological, hepatic, and renal damages. The incidence rate of ATDH (12.4%) was the highest, followed by anemia (8.4%), CHD (5.1%), thrombocytopenia (4.6%), AKI (4.0%), hyperbilirubinemia (3.2%), and leukopenia (2.5%) among the ADRs investigated following TB treatment. The data are shown in Table 7. As a result, subjects with an rs2542670G allele (GA/GG genotype) were associated with increased risk of thrombocytopenia (OR = 3.79, 95% CI = 1.36‐10.52), leukopenia (OR = 5.21, 95% CI = 1.11‐24.37), and chronic kidney damage (OR = 4.26, 95% CI = 1.55‐11.68) following drug administration.

**Table 7:** Association of lnc‐HNF1B‐3:1 polymorphisms and adverse drug reactions of TB patients

Genotypes	Anemia (n = 44)		Thrombocytopenia (n = 24)		Leukopenia (n = 13)		Hyperbilirubinemia (n = 17)		ATDH (n = 65)		AKI (n = 21)		CKD (n = 27)	
	OR (95% CI)	*P*	OR (95% CI)	*P*	OR (95% CI)	*P*	OR (95% CI)	*P*	OR (95% CI)	*P*	OR (95% CI)	*P*	OR (95% CI)	*P*
rs1051838														
GA + GG (n = 356)	0.63 (0.32‐1.24)	.205	0.51 (0.21‐1.23)	.152	0.57 (0.17‐1.89)	.345	0.96 (0.32‐2.85)	.999	0.65 (0.37‐1.15)	.169	1.18 (0.41‐3.43)	.999	0.89 (0.37‐2.15)	.821
AA (n = 170)														
rs12939622														
GA + GG (n = 299)	1.17 (0.60‐2.31)	.733	1.01 (0.42‐2.44)	.999	1.33 (0.38‐4.61)	.764	2.14 (0.67‐6.84)	.289	.80 (0.45‐1.39)	.472	1.48 (0.54‐4.06)	.620	1.45 (0.60‐3.50)	.518
AA (n = 227)														
rs2542670														
GA + GG (n = 253)	1.60 (0.82‐3.13)	.178	**3.79 (1.36‐10.52)**	**.007**	**5.21 (1.11‐24.37)**	**.029**	0.39 (0.12‐1.25)	.120	0.59 (0.32‐1.08)	.099	1.02 (0.39‐2.70)	.999	**4.26 (1.55‐11.68)**	**.003**
AA (n = 273)														
rs1416														
CT + TT (n = 342)	0.90 (0.45‐1.79)	.859	0.57 (0.24‐1.36)	.241	0.92 (0.27‐3.21)	.999	1.47 (0.46‐4.70)	.593	0.73 (0.41‐1.29)	.295	1.78 (0.57‐5.57)	.438	0.98 (0.41‐2.37)	.999
CC (n = 184)														
rs8075185														
CT + TT (n = 382)	1.04 (0.49‐2.21)	.999	0.48 (0.20‐1.16)	.128	0.64 (0.18‐2.23)	.498	1.02 (0.32‐3.27)	.999	1.79 (0.84‐3.80)	.176	0.88 (0.30‐2.54)	.784	0.57 (0.24‐1.36)	.232
CC (n = 144)														
rs2688														
GT + GG (n = 331)	2.06 (0.95‐4.46)	.079	1.99 (0.71‐5.54)	.249	0.50 (0.15‐1.65)	.345	1.22 (0.41‐3.63)	.793	0.75 (0.41‐1.35)	.355	1.13 (0.41‐3.11)	.999	1.75 (0.68‐4.53)	.277
TT (n = 195)														
rs4262994														
CA + AA (n = 374)	0.99 (0.48‐2.07)	.999	0.64 (0.26‐1.59)	.332	0.70 (0.20‐2.44)	.522	5.88 (077‐45.20)	.078	0.76 (0.42‐1.38)	.430	1.353 (0.43‐4.23)	.787	1.48 (0.537‐4.07)	.636
CC (n = 152)														

Note

*P* value has been adjusted for age, gender and BMI; Considering the low frequencies of some minor genotypes, SNPs were stratified based on the dominant model.

## 4 DISCUSSION

Currently, TB is still one of the major threats to human health worldwide. In recent years, several studies, such as case‐control,^20^ family‐based,^21^ candidate gene approaches,^22^ and GWAS,^23^ have explored the association of genetic factors with patient susceptibility to TB. It is widely accepted that approximately 98% of junk DNA is transcribed to non‐coding RNA. However, the roles of lncRNAs in the pathological process of TB remain largely elusive. A previous study demonstrated that the CD244 signaling pathway exhibited a positive correlation with high expression levels of lncRNA‐BC050410 in CD8^+^ T cells stimulated during MTB infection.^24^ Another study demonstrated that the expression levels of two lncRNAs, namely MIR3945HG V1 and MIR3945HG V2 were significantly elevated in the pulmonary tuberculosis patients compared with those noted in the healthy controls.^25^ These findings indicated that lncRNAs could affect the susceptibility of TB by specific mechanisms of action.

In the present study, the potential associations of seven candidate SNPs in the lnc‐HNF1B‐3:1 with the risk of developing TB and with the clinical characteristics of the patients were investigated. The data indicated that the rs2688 of lnc‐HNF1B‐3:1 was possibly associated with the risk of developing TB in the additive model, while rs12939622, rs4262994, and rs2542670 polymorphisms may influence clinical presentations of the disease. This result suggested that genetic variants of the lnc‐HNF1B‐3:1 were associated with susceptibility to TB infection and may function as an important component to TB development.

No significant association was noted with regard to TB susceptibility and the presence of all 7 SNPs of lnc‐HNF1B‐3:1 in the enrolled Western Chinese population, according to single‐locus analysis. Nevertheless, the difference in the genotype distribution of the rs2688 polymorphism in the additive model exhibited a tendency to reach statistical significance. Previous studies revealed significant associations of the minor C allele of rs2688 with decreased levels of fasting insulin and increased risk to type 2 diabetes mellitus.^26^, ^27^ Furthermore, diabetes has been widely recognized as a risk factor for TB development,^28^ which is in accordance with the current results. The results presented in the current study and in previous studies indicated that the GG genotype of rs2688 was not a strong determinant and was considered to confer a weak protection to TB development. Hijikata et al^29^ reported that the AA genotype of the rs1051838 polymorphism was associated with protection against active PTB in younger patients of West African origin. However, the current study failed to demonstrate significant differences in TB analysis, or in further PTB subgroup analysis (data not shown). The differences between the previous studies and the current study were attributed to genetic differences of ethnicity.

Using the clinical data of TB patients, the present study explored whether the 7 candidate SNPs were associated with the clinical features of active TB. The data indicated that the rs12939622 and rs4262994 polymorphisms exhibited a significant contribution to the incidence of fever. Fever is representative of the inflammatory response to MTB infection to a certain degree. The potential TB susceptibility locus rs2688 was not associated with the clinical presentation of the patients. Similarly, the incidence of fever was associated with the rs12939622 and rs4262994 polymorphisms and with genetic predisposition to TB. These results were contradictory and implied that the incidence of TB and its development may be independently affected by different loci. Although the exact mechanism remains unclear, the current study strongly suggested that lnc‐HNF1B‐3:1 polymorphisms and the lnc‐HNF1B‐3:1 genetic locus play important roles in the development of TB.

**Figure 1:**
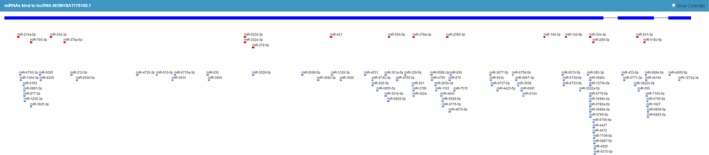
103 miRNAs can bind to lnc‐HNF1B‐3:1

**Figure 2:**
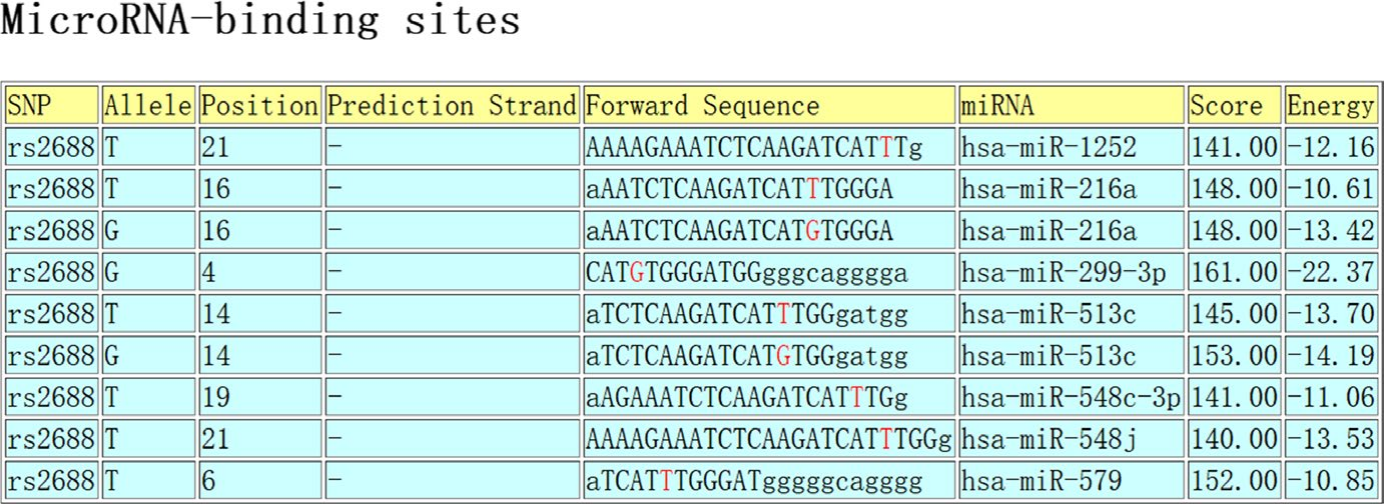
mir‐299‐3p is able to bind with lnc‐HNF1B‐3:1

The investigation of lncRNA function has evolved over the past decades, and one of the compelling hypotheses is the competitive endogenous RNA (ceRNA) hypothesis. This hypothesis proposes that certain RNAs can regulate other transcripts by competing for shared microRNAs.^30^ LncRNAs were reported in an increasing number of studies to act as functional ceRNAs.^31^ With the aid of LNCipedia^8^ and lncRNASNP2,^32^ we demonstrated that 103 miRNAs can bind to lnc‐HNF1B‐3:1 (as shown in Figure 1). Among them, mir‐421,^33^ mir‐142‐3p,^34^ mir‐212‐5p,^35^ and mir‐378a‐3p^36^ were reported to be associated with TB. In addition, mir‐299‐3p was able to bind with lnc‐HNF1B‐3:1 and the binding ability was affected by the mutation of rs2688, as predicted by SNPinfo^37^ and miRNASNP2^38^ (as shown in Figure 2). Therefore, we speculated that rs2688 in lnc‐HNF1B‐3:1 could participate in the development of TB, via influencing the ability of lnc‐HNF1B‐3:1 to interact with candidate miRNAs.

**Figure 3:**
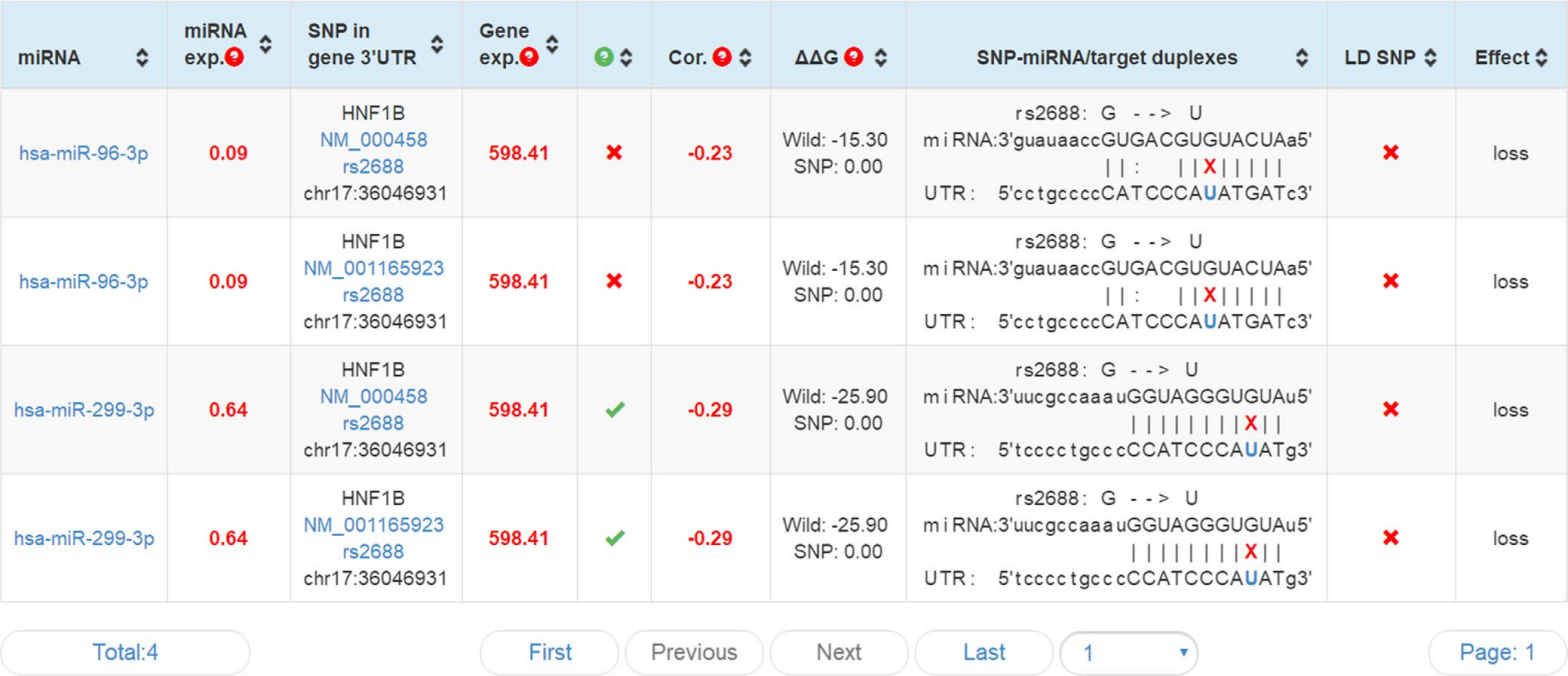
lnc‐HNF1B‐3:1 overlaps the ACACA gene

ADRs can disrupt the treatment of tuberculosis, notably in the long courses of treatment. The most common ADR is liver injury, ranging from 2.55% to 36% as previously reported.^39^, ^40^ In the present study, we reported an incidence of 12.4% in the Western Chinese TB population. To date, PharmaGKB^41^ has provided 27 pairs of annotated variant‐drug pairs for TB that were mainly associated with liver toxicity. Patients with rapid or intermediate acetylator phenotypes of rs1208 in https://www.pharmgkb.org/gene/PA18 may have increased metabolism of isoniazid, as compared to those with slow acetylator phenotypes.^42^ In addition, nephrotoxicity and hematotoxicity are less common but also lethal. The current detection methods lack the identification of appropriate pharmacogenetic loci that can be used as markers for susceptibility. In the present study, we found that individuals with the rs2542670 GA/GG genotypes were associated with an increased risk of thrombocytopenia, leukopenia, and chronic kidney damage following medication. As shown in Figure 3, lnc‐HNF1B‐3:1 overlapped with the *ACACA* gene at one end and the rs2542670 was approximately located at the overlapping area. Acetyl‐CoA carboxylase is an enzyme that is encoded by the *ACACA* gene, which plays a crucial role in the metabolism and biosynthesis of fatty acids and is involved in compound metabolism and various signaling pathways.^43^ Although the specific mechanism is still unclear, the current results provide a certain guide to the clinical application of drug therapy, so as to avoid possible toxicity.

Although the association between lnc‐HNF1B‐3:1 variants and TB susceptibility, TB clinical manifestations and adverse drug reaction were investigated in detail, several limitations were present in the current study. Firstly, the sample size was considerably low, which may lead to false‐positive results. Secondly, the addition of case‐control subjects, such as pneumonia may strengthen the findings obtained. Thirdly, the specific mechanism of TB susceptibility and incidence of lnc‐HNF1B‐3:1 remains unclear and further research is required to offer more insight into this interaction.

In conclusion, the present study identified the G allele of rs2688 in lnc‐HNF1B‐3:1 as a potential TB‐associated allele for a protective effect. In addition, the AA genotype of rs12939622 and rs4262994 appeared to be more prone to susceptibility of the TB subjects to fever, and the rs2542670 GA/GG genotype was found to be related to the increased risk of thrombocytopenia, leukopenia, and chronic kidney damage following medication. The lncRNA HNF1B‐3:1 polymorphisms are promising biomarkers for the evaluation of the patient response to TB infection. However, additional research is required to fully understand the genetic mechanisms of TB and predict an optimal therapeutic patient response.

## CONFLICT OF INTEREST

The authors declare no conflict of interest, financial or otherwise.

## ETHICS APPROVAL AND CONSENT TO PARTICIPATE

Informed consent was obtained from each participant. This study has been approved by the Clinical Trial and Biomedical Ethics Committee of West China Hospital.

## CONSENT FOR PUBLICATION

Not applicable.
